# Earthing as a Supportive Therapy for Post-Spinal Surgery Recovery

**DOI:** 10.3390/jcm14113844

**Published:** 2025-05-29

**Authors:** Paweł Sokal, Maciej Broda, Magdalena Zając, Julia Sokal

**Affiliations:** 1 Department of Neurosurgery, Functional and Stereotactic Neurosurgery, Collegium Medicum, Nicolaus Copernicus University Toruń, 85-067 Bydgoszcz, Poland; maciej.broda@cm.umk.pl; 2 Department of Pedagogy, Casmir Great University, 85-064 Bydgoszcz, Poland; tsf1@wp.pl; 3 Jan Biziel University Hospital nr 2, Collegium Medicum, 85-168 Bydgoszcz, Poland; julkasokal@gmail.com

**Keywords:** spinal surgery, earthing, postoperative pain, creatine kinase

## Abstract

**Background/Objectives**: Spinal surgery often results in injury to the paraspinal muscles and postoperative pain, which is associated with an elevated inflammatory response and increased creatine kinase (CK) levels. Earthing, a practice involving direct or indirect contact with the Earth, facilitates the movement of electric charge between the body and the Earth, thereby stabilizing electrical potentials and influencing biochemical and bioelectrical processes. This study aimed to investigate the effects of earthing on postoperative pain and biochemical parameters. **Materials and Methods**: The study included an earthing group (EG) of 42 patients (18 females) who underwent spinal surgery and were earthed during nighttime postoperative rest. Blood samples were collected to measure serum concentrations of sodium, potassium, urea, glucose, C-reactive protein (CRP), alkaline phosphatase (ALP), calcium, phosphates, CK, iron, ferritin, and transferrin. These parameters were assessed on the day after surgery and the day following earthing. A control group (CG) of 42 patients (25 females) who underwent surgery for lumbar spondylosis did not receive earthing. **Results**: The median reduction in the EG was significantly greater than in the CG (for CK 45.0 and 20.0 U/L; for ALP 6.0 and 1.0; for transferrin 0.17 and 0.08, respectively). The median CRP difference in the EG was 0.05 mg/dL, significantly lower than in the CG, 17.2 mg/dL. The median reduction in pain intensity in VAS score was greater in the EG–2.0 compared to the CG-1.0, acknowledging a strong analgesic effect of earthing (*p* < 0.01). **Conclusions**: Earthing after spinal surgery seems to promote recovery by reducing inflammation and pain, and accelerating general healing, suggesting its potential as a supportive postoperative therapy.

## 1. Introduction

Spinal surgery is associated with injury to the paraspinal muscles. The extent of muscle injury correlates with the type of surgery performed, such as microdiscectomy, endoscopic discectomy, micro decompression, or percutaneous spinal fusion. Minimally invasive methods generally result in less muscle injury and reduced postoperative pain, though these effects are not always conclusive [1,2]. It has been confirmed that serum creatine kinase (CK) levels are an indicator of muscle damage following spinal surgery [3,4]. CK is typically elevated in medical conditions such as myocardial infarction, muscular dystrophy, and cerebral diseases. Significant correlations have been found between the extent of spinal surgery—the number of levels fused and duration of surgery—and CK values [5]. Increased serum CK levels have also been linked to higher levels of postoperative pain. Spinal surgery is also associated with a certain degree of blood loss and destruction of spinal bone tissue. Direct contact with the Earth, whether barefoot or indirectly via a cable connected to a conductive system like a mat, sheet, or ankle band, establishes electrical equilibrium with the Earth. This connection influences bioelectrical processes and alters physiological parameters [6,7]. During earthing (grounding), charges of less than 10 nA flow between the human body and the ground in a charge-discharge cycle associated with movement [6,8]. The potential of the human body during movement with an established ground path is near zero, whereas when the ground path is interrupted, the potential fluctuates [6]. Earthing has been shown to reduce stress, improve sleep, alter cortisol excretion rhythms, reduce blood viscosity, modulate the autonomic nervous system, and heart rate variability [7]. Additionally, earthing could reduce symptoms of muscle damage and soreness after eccentric muscle contractions, as indicated by CK elevation [9]. Earthing has been identified as a potential anti-inflammatory factor that may influence calcium and phosphate homeostasis, as well as iron metabolism [10]. Studies have reported significant reductions in serum concentrations of sodium, potassium, magnesium, phosphate, calcium, iron, total protein, and albumin, alongside increases in transferrin, ferritin, and globulin fractions (α1, α2, β, and γ). A reduction in renal excretion of calcium and phosphorus was also observed. Furthermore, earthing was associated with notable effects on thyroid hormones, cortisol levels, and the metabolism of glucose and urea, particularly in the context of physical exercise [10,11,12,13]. Earthing therapy has been shown to accelerate wound healing in individuals with diabetes and to improve blood circulation, primarily through reduced blood viscosity and enhanced transport of nutrients and oxygen [14,15,16]. Evidence supporting decreased blood viscosity includes studies demonstrating reduced red blood cell aggregation following earthing therapy [17]. This study aimed to investigate changes in selected biochemical parameters in patients after lumbar spinal surgery and to determine whether earthing during postoperative nighttime relaxation impacts the recovery.

## 2. Materials and Methods

A prospective study was conducted in the Department of Neurosurgery, Functional and Stereotactic Neurosurgery at Jan Biziel University Hospital No. 2 in Bydgoszcz on 84 patients who underwent surgery for lumbar spondylosis. Participants were assigned to study groups using randomization methods. Two approaches were employed: simple first-degree randomization, in which each participant had an equal chance of being allocated to any group (achieved using a coin toss), and block randomization, which was used to maintain an equal number of participants in both the intervention and control groups. Clinical research was conducted in accordance with established ethical standards to ensure maximum safety for participants and to uphold their rights. Each participant provided written informed consent prior to enrollment and was given detailed information regarding the study. The investigator responsible for enrolling participants was required to explain all aspects of the study clearly, addressing any uncertainties or concerns raised by the participant. Ethical approval (Nr 197/2020) was obtained from the Ethics Committee at the Collegium Medicum of the Nicolaus Copernicus University in Bydgoszcz, Poland, in accordance with the Declaration of Helsinki. Inclusion criteria were: qualification for surgical intervention of the lumbar spine and signed informed consent to participate in the study. Exclusion criteria were: diagnosed neoplastic disease and hemostatic disorders, pregnancy, and lack of signed informed consent. Patients were qualified for decompressive lumbar surgery. Most procedures were similar in terms of invasiveness, as the majority of patients underwent single-level lumbar spine surgery. Surgical approaches were either unilateral or bilateral. Microdecompression was commonly performed and included fenestration of the ligamentum flavum, partial medial facetectomy, foraminotomy, central flavectomy, hemilaminectomy, or laminectomy; laminectomy was always performed using a bilateral approach. Interspinous stabilization was also performed using a bilateral approach and was minimally invasive.

All patients were examined in the same manner. Blood samples were collected to measure serum concentrations of sodium, potassium, urea, glucose, C-reactive protein (CRP), alkaline phosphatase, calcium, phosphates, creatine kinase (CK), iron, ferritin, and transferrin. Analyses of CK and CRP were conducted in 84 patients, while additional biochemical parameters were evaluated in a subset of 56 patients.

These measurements were taken on the first day after surgery and on the second day after surgery. The effectiveness of earthing (grounding) was confirmed using an electrometer, which demonstrated a reduction in electrical potential following activation of the grounding system. Biochemical analyses were performed using the Cobas Integra 400 Plus Analyzer (Roche Diagnostics GmbH, Mannheim, Germany), employing validated assays and calibrated procedures for the measurement of CRP, iron, phosphate, and CK levels.

**Figure 1:**
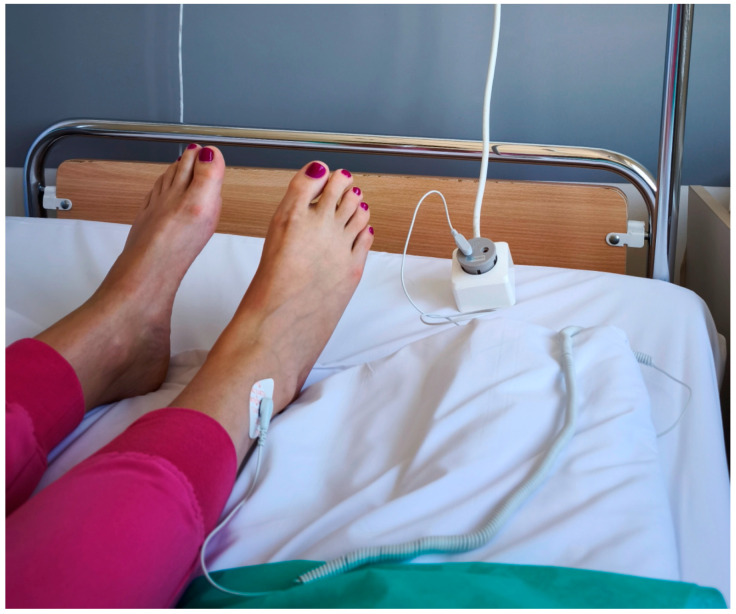
The experimental earthing setup and the sham earthing setup (with no internal wire connections in the socket) appeared identical in appearance.

Participants were divided into two groups:

Earthing group (EG) (*n* = *42*): Patients were grounded via an adhesive electrode pad attached to the ankle and connected to the earthing system of an electrical socket Figure 1. Patients in the study group were earthed during nighttime rest between the first and the second day after surgery. Pain was assessed, and blood was collected and analyzed on the day after surgery and on the first and second mornings following surgery. Thirty-eight patients underwent surgery on one segment of the lumbar spine, and four patients underwent surgery on two segments of the lumbar spine. The most frequent was the L4/L5 level (24 patients), next the L5/S1 level (15 patients), and the L3/L4 level was operated on six patients.

Control group (CG) (*n* = *42*): Patients received a sham intervention where the electrode pad was applied but not connected to the earthing system. The CG comprised patients who underwent similar surgeries for lumbar spondylosis. Thirty-six patients were operated on at one level of the lumbar spine, and six patients on two levels. The most frequent was the L4/L5 level (27 patients), ten at the L5/S1 level, and nine at the L3/L4 level. Patients in the CG did not undergo earthing; however, their blood samples were collected and analyzed using the same protocol on the first and second mornings following surgery. Pain was also assessed at the same time points.

The average age in the study group was 53 ± 14 years, while the CG had an average age of 60 ± 11 years. The intensity of pain was assessed using the Visual Analogue Scale (VAS) on the first day after surgery and on the second day after surgery, following a night of earthing in the EG and a night of sham-earthing in the CG. All patients were administered a uniform analgesic regimen comprising paracetamol.

### Statistical Analysis

In the statistical analysis, frequency distributions, descriptive statistics, and significance tests were utilized. The chi-squared test was employed to examine differences between the demographic characteristics of the subjects. The analysis was conducted using the Statistica software version 13 package by Tibco Software Inc., Palo Alto, CA, USA; statistical significance was assumed for tests meeting the condition of *p* < 0.05. For differences between groups in ordinal variables (VAS) or quantitative variables (biochemical indices and differential indices levels), the Mann–Whitney U test was used. For differences between measurements, the Wilcoxon test was applied. The choice of non-parametric tests was dictated by the sample sizes and the lack of normality in the distributions of most variables (verified using the Kolmogorov–Smirnov test). In the conducted study, effect size was calculated using the rank-biserial correlation coefficient (*r*_c_) for matched pairs, derived from the Mann–Whitney or Wilcoxon test. The formula for this coefficient is appropriate for nonparametric comparisons. Effect sizes for selected outcomes: VAS (line 120): EG *r* = 0.60 (strong), CG *r* = 0.52 (strong); CK (line 129): EG *r* = 0.55 (strong), CG *r* = 0.37 (moderate); CRP (line 136): EG *r* = 0.16 (small), CG *r* = 0.59 (strong); ALP (line 142): EG *r* = 0.30 (small), CG *r* = 0.13 (small); Phosphates (line 151): EG *r* = 0.03 (small), CG *r* = 0.26 (small); Calcium: EG *r* = 0.25 (small), CG *r* = 0.08 (negligible); Transferrin: EG *r* = 0.33 (moderate), CG *r* = 0.12 (small); Ferritin: EG *r* = 0.09 (small), CG *r* = 0.25 (small); Iron: EG *r* = 0.21 (small), CG *r* = 0.005 (negligible)

## 3. Results

### 3.1. Effect of Earthing on Postoperative Pain (N = 84)

**Figure 2:**
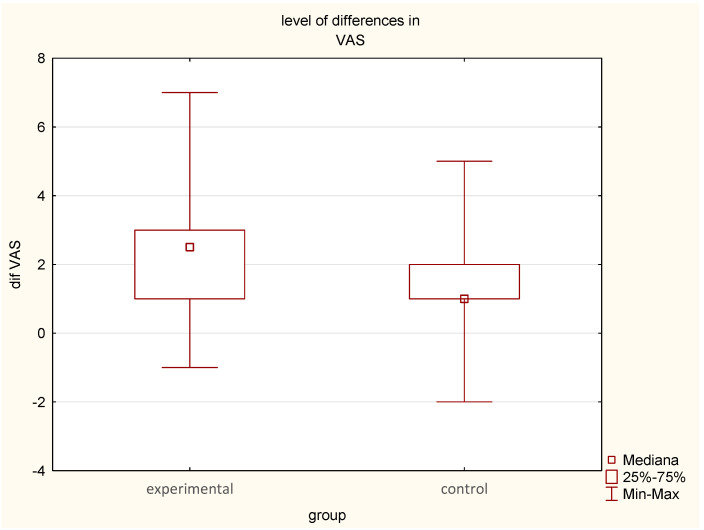
Differences in intensity of pain in VAS before and after earthing or sham-earthing between the experimental and control groups, *p* < 0.01.

- On postoperative day 1, mean VAS scores were 7.14 ± 1.41 (median = 7) in the EG and 6.6 ± 1.95 (median = 7) in the CG.
- On postoperative day 2, pain significantly decreased in the EG to 4.64 ± 1.29 (median = 5), *p* < 0.05, compared to 5.26 ± 1.47 (median = 5) in the CG, *p* < 0.05.
- The mean reduction in pain intensity was greater in the EG (2.50 points) (median = 2.0) compared to the CG (1.59 points) (median = 1.0), suggesting a meaningful analgesic effect of earthing in the experimental group (Figure 2, Table 1, Table 2 and Table 3). The difference in change in pain intensity between the two groups was statistically significant (*p* < 0.01)

### 3.2. Effect of Earthing on Postoperative Muscle Injury (N = 84)

- Preoperative CK levels in the EG were 311.97 U/L (median = 208.5), decreasing to 257.36 U/L (median = 182.5) after one night of earthing (*p* < 0.0001).
- In the CG, CK levels declined from 373.45 U/L (median = 307) to 332.95 U/L (median = 229.5) (*p* < 0.001).
- The mean CK reduction in the EG (54.61 U/L) (median = 44.5 U/L) was greater than in the CG (40.5 U/L) (median = 26 U/L), indicating accelerated muscle recovery. However, the difference between the two groups was not significant. (Table 1, Table 2 and Table 3).

### 3.3. Effect of Earthing on Inflammatory Markers (N = 84)

**Figure 3:**
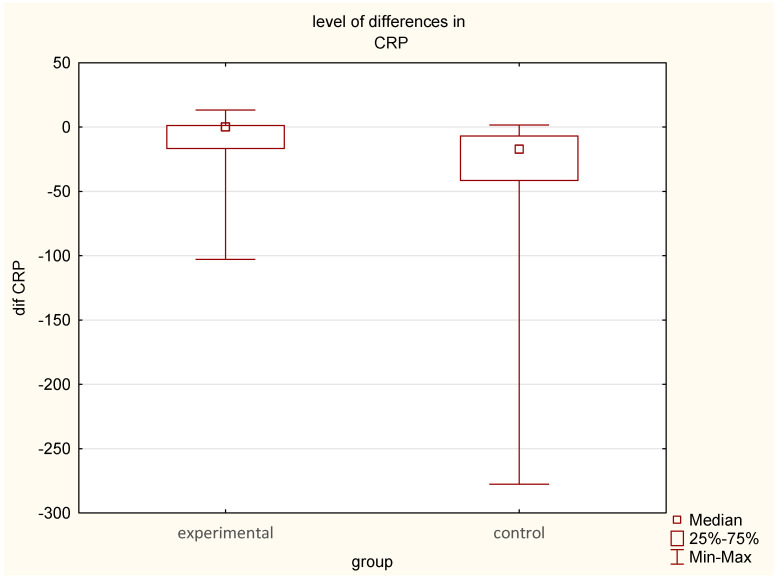
Comparison of pre- and post-intervention differences in CRP levels between the experimental group (earthing) and the control group (sham-earthing), *p* < 0.01.

- Postoperative CRP levels increased significantly in both groups, but the rise was lower in the EG (15.34 mg/L to 24.42 mg/L) compared to the CG (24.54 mg/L to 57.42 mg/L).
- The median CRP difference in the EG was 10.07 mg/dL, significantly lower than the CG 33.39 mg/dL (*p* < 0.001), suggesting a dampened inflammatory response in earthed patients (Table 1, Table 2 and Table 3, Figure 3).

### 3.4. Effect of Earthing on Calcium-Phosphate Metabolism (N = 56)

**Figure 4:**
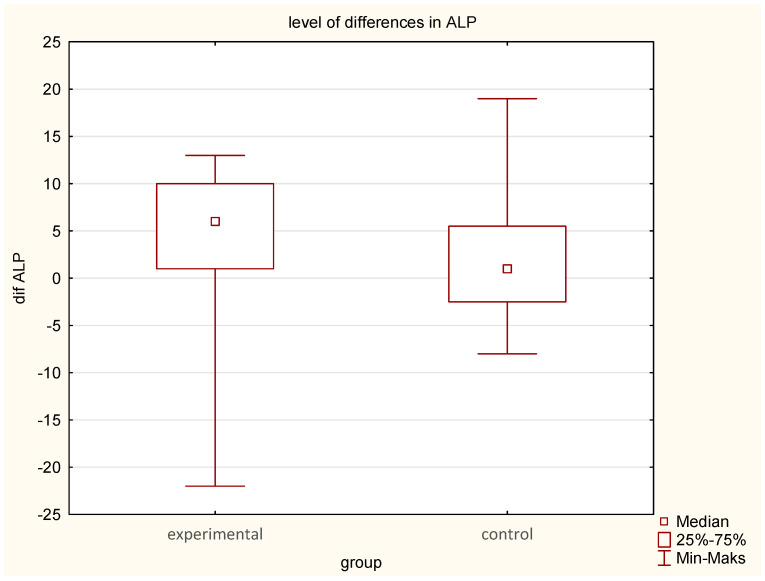
Comparison of pre- and post-intervention differences in ALP levels between the experimental group (earthing) and the control group (sham-earthing), *p* = 0.02.

**Figure 5:**
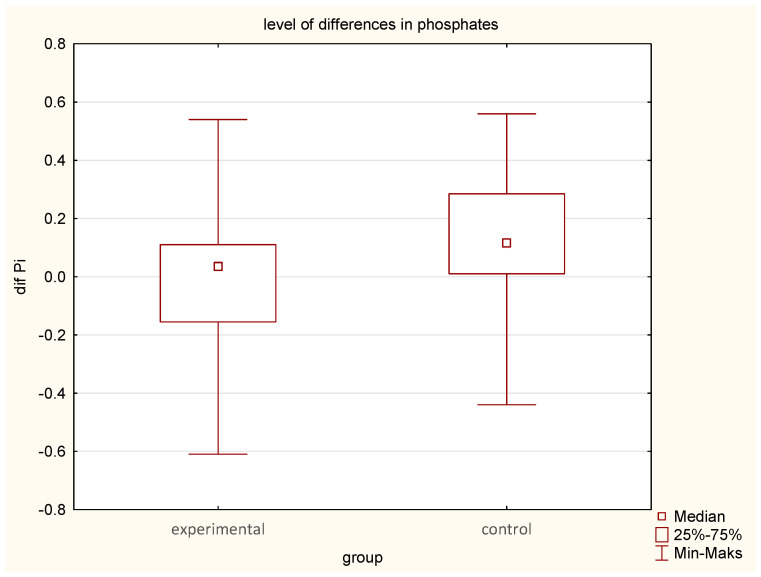
Comparison of pre- and post-intervention differences in phosphate levels between the experimental group (earthing) and the control group (sham-earthing), *p* = 0.02.

- ALP decreased significantly in the EG from 65.5 U/L to 61.4 U/L; (median difference—6 U/L, compared to a smaller reduction in the CG (66.6 U/L to 65.1 U/L), (median difference—1.0) (*p* = 0.02). (Table 1, Table 2 and Table 3; Figure 4).
- Phosphate levels increased significantly in the EG (*p* = 0.02), whereas calcium alterations were not statistically significant (Table 1, Table 2 and Table 3; Figure 5).

### 3.5. Effect of Earthing on Iron Metabolism (N = 56)

**Figure 6:**
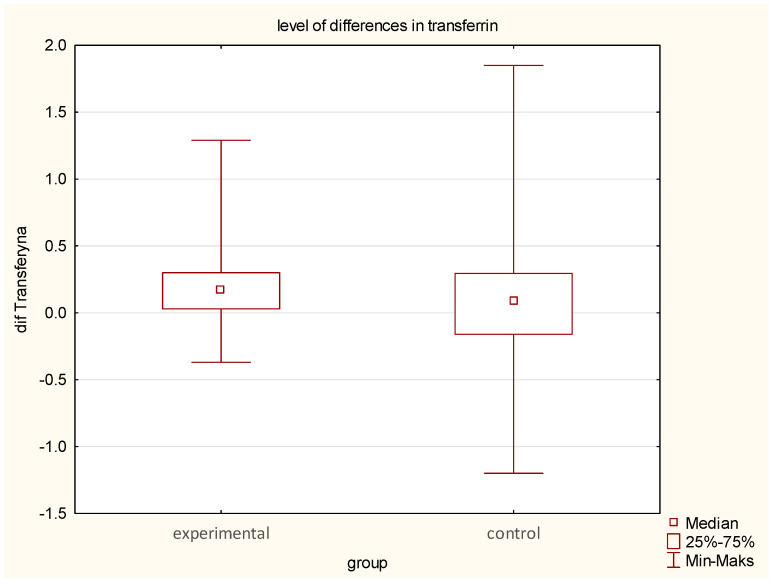
Comparison of pre- and post-intervention transferrin alterations between the experimental group (earthing) and the control group (sham-earthing).

- Transferrin levels declined more significantly in the EG (from 2.46 to 2.26 g/L) (median difference—0.17, *p* = 0.02) than in the CG (from 2.28 to 2.24 g/L) (median difference—0.08).
- Ferritin variability was greater in the EG (145 ng/mL—no median change), with both increases and decreases, whereas the CG exhibited a more uniform rise (from 131 to 143 ng/mL) (*p* = 0.02).
- Median Iron increased in EG from 40.5 to 48.8 µg/dL (*p* = 0.05) while in CG decreased from 43.5 to 34.5 µg/dL (*p* = 0.96) (Table 1, Table 2 and Table 3; Figure 6).

## 4. Discussion

This study highlights the potential benefits of earthing (grounding) in postoperative recovery following spinal surgery. The study demonstrated a significant reduction in pain intensity on the second day after surgery in patients who were earthed during the second night after surgery in comparison to patients who were not earthed. Patients who undergo spinal surgery often experience postoperative pain, which is associated with injury to the paraspinal muscles. The severity of this pain is related to the degree of surgical invasiveness [1,18]. Some studies suggest the analgetic effect of earthing; however, there is no scientific evidence [7,19]. In our study, the median visual analog scale (VAS) score was 7; however, patients who were earthed on the first postoperative night following lumbar surgery experienced greater pain relief. Minimally invasive procedures, such as endoscopic discectomy or microdecompression, are associated with minimal tissue damage, less muscle retraction, and lower postoperative pain [2]. Not all of our procedures met the criteria for minimally invasive surgery, particularly those involving two spinal segments. Extensive muscle damage is confirmed by elevated serum CK levels and severe postoperative pain [5]. Highly invasive surgery results in the highest postoperative to preoperative CK ratio, while minimally invasive surgery results in the lowest [20]. The CK enzyme is commonly used as an objective indicator of delayed onset muscle soreness (DOMS), which is closely associated with muscle damage [21]. In our study, a greater reduction in CK levels was observed on the second postoperative day in patients who underwent earthing during the second postoperative night, compared with those who were not earthed. The median decrease in CK levels was greater in the EG than in the CG. However, despite a larger sample size in the EG (n = 42), statistical significance was not reached. It is likely that a narrower distribution of individual results would have increased the likelihood of reaching statistical significance. These findings align with previous reports demonstrating that grounding reduces inflammation, modulates metabolism and immune function, and accelerates recovery from delayed-onset muscle soreness (DOMS), though without significant reductions in pain intensity [9,10,11,12,13,22,23,24]. Based on the results of a pilot study conducted in 2010, the authors observed that grounding the body to the earth reduces markers of inflammation, modulates immune system activity, and accelerates recovery from DOMS, as indicated by faster reduction in creatine kinase levels [9]. This phenomenon could be attributed to the anti-inflammatory effects of negative charge supply and electron participation. Brown et al. identified markers of earthing in correlations between white blood cells, bilirubin, creatine kinase, and inorganic phosphorus among grounded subjects [24]. Participants who were not grounded reported higher pain perception associated with DOMS in the same study. In a study conducted by Pantoja et al. in 2009, levels of creatine kinase (CK) were measured in participants performing resistance exercises in water and on land. It was observed that individuals who were grounded in water showed a decrease in CK levels 24 h after exercise, with a further decrease observed 48 h post-exercise. In contrast, participants who exercised on land showed an increase in CK levels [25]. The smaller difference in inflammatory markers between the second and third postoperative days in individuals who were earthed during the second night suggests a potentially attenuated inflammatory response to surgical injury, compared to controls who were not earthed. This is supported by a significantly smaller increase in CRP levels observed in the EG. Effect sizes were clinically meaningful for the VAS and CK parameters, indicating substantial changes in both studied groups. A strong effect size was observed for CRP in the CG, suggesting that the inflammatory process remained unaffected. It is important to note that surgical trauma typically induces a CRP elevation, with peak levels occurring between the second and third day postoperatively, most commonly on the second day [26,27]. Earthing significantly impacts various physiological processes; an 8-h grounding session can notably alter calcium-phosphate homeostasis, reducing ionized calcium and inorganic phosphorus levels and affecting iron metabolism, leading to decreased iron levels and increased levels of transferrin and ferritin. Additionally, earthing reduces concentrations of sodium, potassium, and magnesium, while promoting an increase in globulins A1, A2, B, and G [10]. Earthing after exercise alters protein metabolism during recovery, leading to changes in blood urea and creatinine levels [12]. Earthing reduces the cardinal signs of inflammation following injury, leading to the resolution of symptoms such as swelling, redness, and pain [28]. Müller et al. demonstrated that earthing during nighttime rest promotes muscle recovery following intense eccentric muscle loading from exercise. Markers of inflammation, such as IP-10, MIP-1α, sP-Selectin, and CK, were more pronounced in athletes who did not undergo earthing compared to those who were earthed. Earthing during nighttime sleep reduced inflammation symptoms and contributed to a lesser increase in CK levels induced by intensive eccentric muscle loading in grounded participants [22]. These findings align with the results of our study, which demonstrate accelerated recovery from surgical injury when patients undergo earthing during sleep. This is evidenced by reduced CK levels, a smaller increase in CRP, an increase in phosphate concentrations, and normalization of calcium-phosphate metabolism leading to the healing of bones injured during surgery, as well as diminished pain perception in earthed patients. Prior studies have suggested that earthing can mitigate oxidative stress by providing electrons that attenuate free radicals, thereby reducing tissue damage and promoting healing [7]. Earthing provides an electric charge that helps neutralize the detrimental reactions of free radical species associated with the inflammatory process [7,28]. Earthing might be seen as an important factor promoting the healing of muscle soreness and damage following spinal surgery in rehabilitated patients, utilizing the same mechanisms that enhance acute and long-term recovery after intensive exercises [22]. In our study, the increase in serum iron concentration, along with a decrease in transferrin and stable ferritin levels in the experimental group, may suggest an enhanced readiness for iron utilization in response to postoperative blood loss. In this report, earthing was associated with a reduction in alkaline phosphatase levels, consistent with previous findings. However, the effect on inorganic phosphate levels differed; whereas earlier studies reported a decrease after 1 to 8 h of earthing, our results showed a slight elevation [10]. Phosphate concentrations normally tend to decrease following, e.g., gastrointestinal surgery [29]. These alterations observed in patients following spinal surgery warrant further investigation. The credibility of our study is strengthened by the use of a sham-earthing setup, which was identical in appearance (Figure 1) and installation to the true earthing system, thereby minimizing the placebo effect. The only difference was the disconnection of the electrical circuit via a closed switch housed within a plastic enclosure, effectively preventing grounding while maintaining blinding. In conclusion, earthing following spinal surgery may support recovery from iatrogenic injury to paraspinal muscles and spinal structures, as suggested by a reduced inflammatory response and trends toward normalization in creatine kinase, calcium-phosphate, and iron metabolism. These findings warrant further investigation in larger, controlled studies.

### Limitations

The changes in biochemical parameters were minor and not clearly distinguishable. The relatively small sample size and the use of single-time-point observations following the earthing intervention limit the interpretability of the results. Additionally, the study population was heterogeneous due to the inclusion of various types of lumbar spine surgeries, such as fenestrations, laminectomies, and discectomies, each involving varying degrees of bone and soft tissue disruption. However, in the majority of cases, the overall level of surgical invasiveness was comparable. The clinical application of earthing remains a relatively underexplored and somewhat controversial area, with only a limited number of high-quality studies available. In this context, we considered earthing as a potential complementary intervention rather than a primary treatment modality. Future studies with larger, more homogeneous groups, extended follow-up periods, and more comprehensive data collection would likely yield clearer outcomes and help reveal statistical trends, thereby facilitating interpretation.

## 5. Conclusions

Earthing appears to be a supportive intervention that reduces postoperative pain and mitigates the inflammatory response following spinal surgery, reflected by a lower increase in CRP levels among earthed patients. The observed impact on calcium-phosphate and iron metabolism suggests that earthing may influence broader physiological processes. Reduced ALP levels in the earthed group may indicate improved bone turnover, while changes in transferrin and ferritin levels may reflect favorable, altered iron homeostasis. These findings support the need for further research into the potential integration of earthing into postoperative protocols to enhance recovery and improve patient outcomes

## Figures and Tables

**Table 1:** Results of the non-parametric analysis with the Mann–Whitney U test, presenting the sum of ranks of measured parameters before and after earthing, evaluated in both the earthing and control groups. U—compares the ranks of values from two independent samples. Z—a standardized version of the U.

Parameter	U Mann-Whitney Test. Statistical Significance *p* < 0.05000									
	Sum.of Ranks exp EG	Sum of Ranks contr CG	U	Z	*p*	Z cor	*p*	N.exp	N.contr	*p*
VAS before	1843.5	1726.5	823.5	0.51887	0.603851	0.53562	0.592218	42	42	0.602522
VAS after	1554	2016	651	−2.06207	0.039202	−2.1475	0.031755	42	42	0.038749
Na [mmol/L] before	791.5	804.5	385.5	−0.09832	0.921677	−0.09927	0.920922	28	28	0.915857
Na [mmol/L] after	893.5	702.5	296.5	1.55675	0.119531	1.56704	0.117106	28	28	0.118157
K [mmol/L] before	737	859	331	−0.9914	0.321489	−0.99174	0.321324	28	28	0.324159
K [mmol/L] after	726	870	320	−1.17166	0.241335	−1.17198	0.241206	28	28	0.243438
urea [mg/dL] before	838.5	757.5	351.5	0.65547	0.512163	0.65623	0.51168	28	28	0.509859
urea [mg/dL] after	815	781	375	0.27038	0.786866	0.27067	0.786645	28	28	0.788512
glucose [mg/dL] before	833	763	357	0.56535	0.571839	0.56552	0.571721	28	28	0.574634
glucose [mg/dL] after	681	915	275	−1.90907	0.056254	−1.91103	0.056002	28	28	0.05581
CRP [mg/L] before	1574.5	1995.5	671.5	−1.87867	0.06029	−1.8788	0.060273	42	42	0.059425
CRP [mg/L] after	1479.5	2090.5	576.5	−2.72855	0.006362	−2.72863	0.00636	42	42	0.005849
ALP [U/L] before	790	806	384	−0.1229	0.902185	−0.12294	0.902152	28	28	0.90297
ALP [U/L] after	731.5	864.5	325.5	−1.08153	0.279462	−1.08249	0.279034	28	28	0.278221
Ca [mmol/L] before	815.5	780.5	374.5	0.27858	0.78057	0.2788	0.780398	28	28	0.776022
Ca [mmol/L] after	760.5	835.5	354.5	−0.60631	0.544307	−0.60699	0.54386	28	28	0.541771
Pi [mmol/L] before	678.5	917.5	272.5	−1.95003	0.051173	−1.95107	0.05105	28	28	0.049667
Pi [mmol/L] after	847	749	343	0.79476	0.426753	0.79503	0.426595	28	28	0.429735
CK [U/L] before	1612.5	1957.5	709.5	−1.53872	0.123873	−1.53878	0.12386	42	42	0.123207
CK [U/L] after	1569	2001	666	−1.92788	0.053871	−1.92794	0.053863	42	42	0.053555
Fe [ug/dL] before	821	775	369	0.3687	0.712349	0.36883	0.712255	28	28	0.714479
Fe [ug/dL] after	882	714	308	1.3683	0.171219	1.3687	0.171094	28	28	0.172551
Ferritin [ng/mL] before	822.5	773.5	367.5	0.39328	0.69411	0.3933	0.6941	28	28	0.69034
Ferritin [ng/mL] after	820	776	370	0.35232	0.724601	0.35234	0.724587	28	28	0.726657
Transferin [g/L] before	814	782	376	0.254	0.799499	0.25403	0.799472	28	28	0.801056
Transferin [g/L] after	771	825	365	−0.43425	0.664106	−0.43435	0.664036	28	28	0.666507

**Table 2:** Postoperative changes in pain and biochemical parameters before and after earthing were evaluated in both the earthing and control groups, including comparisons of means, medians, and standard deviations.

Differences	Group	N	Mean	Median	Minimum	Maximum	Std.dev
dif VAS	ex	42	2.5	2.5	−1	7	1.62676
dif Na	ex	28	−0.9286	−100.000	−8.000	40.000	272.068
dif K	ex	28	433.214	0.00000	0.000	1010.000	5086.806
dif urea	ex	28	40.357	450.000	−7.000	190.000	600.914
dif GLU	ex	28	372.143	3450.000	−24.000	1570.000	3660.435
dif CRP	ex	42	−10.0762	0.05	−102.9	13.2	24.16638
dif ALP	ex	28	41.429	600.000	−22.000	130.000	819.085
dif calcium	ex	28	0.0400	0.04000	−0.110	0.1800	0.07832
dif phosphate	ex	28	−0.0296	0.03500	−0.610	0.5400	0.24606
dif CK	ex	42	54.619	44.5	−128	250	69.79131
dif iron	ex	28	−93.571	−600.000	−58.000	570.000	2463.393
dif ferritin	ex	28	39.286	350.000	−85.000	1410.000	4528.096
dif transferrin	ex	28	0.2046	0.17000	−0.370	12,900	0.35132
dif VAS	ctr	42	1.5952	1	−2	5	1.53113
dif NA	ctr	28	0.2500	0.0000	−6.000	60.000	257.660
dif K	ctr	28	438.571	40.000	0.000	1010.000	5,043,640
dif urea	ctr	28	35,357	30.000	−6.000	150.000	522.446
dif GLU	ctr	28	276.429	180.000	−38.000	1340.000	3894.366
dif CRP	ctr	42	−33.3857	−17.2	−277.6	1.6	48.93226
dif ALP	ctr	28	15.357	10.000	−8.000	190.000	570.563
dif CA	ctr	28	0.0164	0.0200	−0.130	0.2600	0.09318
dif phosphate	ctr	28	0.1143	0.1150	−0.440	0.5600	0.24990
dif CK	ctr	42	40.5	26	−295	325	96.83661
dif iron	ctr	28	−0.4643	−20.000	−51.000	370.000	1778.781
dif ferritin	ctr	28	−97.857	−120.000	−84.000	700.000	2725.919
dif transferrin	ctr	28	0.0943	0.0850	−1.200	18.500	0.55129

**Table 3:** Statistical significance of differences between postoperative parameters before and after earthing in the earthing group (EG) and in the control group (CG). Statistically significant values of differences of VAS, CRP, phosphates, and ferritin are highlighted in bold. U—compares the ranks of values from two independent samples. Z—a standardized version of the U.

Differences in Parameters	Sum of Ranks EG	Sum of Ranks CG	U	Z	*p*
dif VAS	2071.500	1498.500	595.500	2.558574	**0.010511**
dif NA	705.000	891.000	299.000	−1.516	0.129575
dif K	787.500	808.500	381.500	−0.164	0.889223
dif urea	819.000	777.000	371.000	0.336	0.736924
dif GLU	867.500	728.500	322.500	1.131	0.258186
**dif CRP**	2249.500	1320.500	417.500	4.150972	**0.000033**
**dif ALP**	**936.500**	**659.500**	**253.500**	**2.261**	**0.023736**
dif Calcium	869.000	727.000	321.000	1.155	0.247980
**dif phosphate**	**656.500**	**939.500**	**250.500**	**−2.311**	**0.020859**
dif CK	1855.000	1715.000	812.000	0.621751	0.534106
dif iron	703.500	892.500	297.500	−1.540	0.123473
**dif ferritin**	**918.500**	**677.500**	**271.500**	**1.966**	**0.049251**
dif transferrin	854.000	742.000	336.000	0.90947	0.363103

## Data Availability

DATA are available on demand.

## Abbreviations

The following abbreviations are used in this manuscript:

DOMS	Delayed onset muscle soreness
CK	creatine kinase
CRP	C—reactive protein
